# A varicocoele mimicking a hydrocoele in a man with portal hypertension: a case report

**DOI:** 10.1186/1752-1947-2-363

**Published:** 2008-12-04

**Authors:** George Yardy, Akkib Rafique, Iain Sellers, Lawrence Berman, Nigel Bullock

**Affiliations:** 1 Department of Urology, Addenbrooke's Hospital, Cambridge, UK; 2 Department of Radiology, Ealing Hospital, London, UK; 3 Department of Radiology, Addenbrooke's Hospital, Cambridge, UK

## Abstract

**Introduction:**

Hydrocoele is a condition frequently encountered in adult urological practice. It is usually of benign aetiology and often diagnosed on clinical grounds. Surgical repair, if indicated, is generally straightforward.

**Case presentation:**

We report a 53-year-old man with liver cirrhosis and clinical features of a hydrocoele, in whom flow was demonstrated using Doppler ultrasonography in the fluid around the testis, which communicated via varices with the left renal vein.

**Conclusion:**

In this patient with misleading clinical signs, diagnosis was established radiologically. Had surgery proceeded without this investigation, significant intra-operative bleeding would have been likely.

## Introduction

A hydrocoele causes fluctuant non-tender unilateral scrotal swelling which is irreducible and may be tense or lax. It is caused by an abnormal quantity of fluid within the tunica vaginalis. In adults, it is usually idiopathic, but may be secondary to trauma, infection, neoplasia or lymphatic obstruction. Paediatric hydrocoele is usually associated with a patent processus vaginalis. A careful history and clinical examination usually establishes the diagnosis and further investigations are not always required. Treatment is often not offered unless the condition troubles the patient particularly. Hydrocoele repair is, however, a frequently performed relatively minor procedure. We present a patient with a hydrocoele on clinical grounds, in whom further radiological investigation demonstrated that the fluid surrounding the testis was blood within a scrotal varix which had developed as a result of a portal-systemic anastomosis involving the splenic and left renal veins.

## Case presentation

A 53-year-old man was referred for assessment of a left scrotal swelling which was slightly uncomfortable and had been particularly noticeable for 3 months. He had a history of non-insulin-dependent diabetes mellitus and hepatic cirrhosis, which was probably secondary to non-alcoholic steatohepatitis. He had oesophageal varices which were asymptomatic and were being monitored. He was examined in the outpatients department and the clinical notes recorded that, "what he appears to have is a lax left hydrocoele which is absolutely typical and of textbook nature."

**Figure 1:**
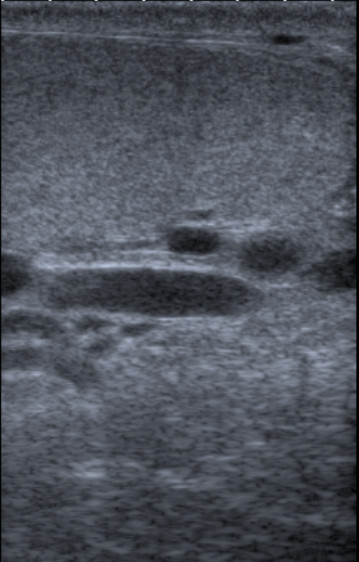
Ultrasound image – left testis focally normal.

**Figure 2:**
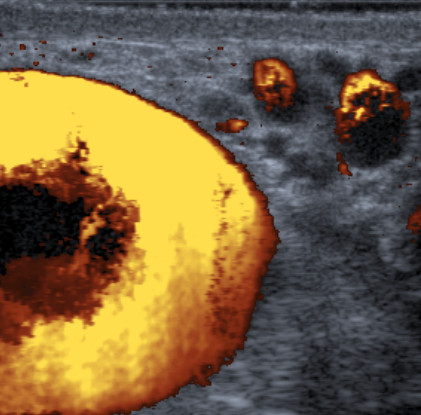
Colour Doppler ultrasound image – flow demonstrated in fluid surrounding left testis.

**Figure 3:**
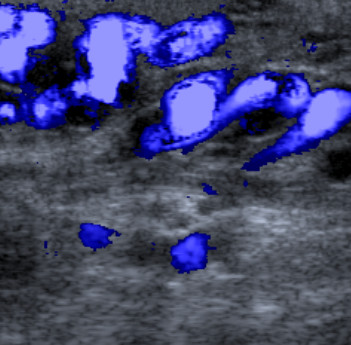
Colour Doppler ultrasound image – variceal gonadal vein in left inguinal canal.

As the testis was difficult to feel within the fluid collection, a scrotal ultrasound (US) examination was arranged to exclude a testicular tumour as the source of the hydrocoele. This showed no focal lesion within either testis (Figure 1). However, Doppler ultrasonography revealed flow within the fluid surrounding the left testis (Figure 2), communicating via a large scrotal varicocoele with large varices. Figure 3 shows flow demonstrated within these dilated vessels which could be followed through the left inguinal canal (Figure 3) to the region of the left renal vein. Extensive spleno-renal varices were recorded.

Conservative treatment was advised as a result of this investigation.

## Discussion

Unusual portal-systemic shunts in portal hypertension have been recorded including communication between a coronary vein varicocoele and patent umbilical vein, superior mesenteric vein and inferior vena cava, splenic vein and abdominal wall, spleno-retroperitoneal and omphalo-ilio-caval anastomosis [1]. Large scrotal varicocoeles secondary to portal hypertension have been described [2,3].

Our patient had cirrhosis and a unilateral scrotal swelling which appeared on clinical grounds to be a hydrocoele but US examination established that it was a varicocoele. It is a noteworthy case because the varicocoele did not have the typical "bag of worms" appearance of multiple varicosities within the hemiscrotum: there was actually a solitary shunt vessel enveloping the testis. This vein could be traced along the inguinal canal and into the abdomen, communicating with abnormal dilated vessels arising from the spleen. We report an unusual form of spleno-renal shunt.

## Conclusion

Attempted hydrocoele repair in this patient would have resulted in marked unanticipated blood loss and sudden ligation of the scrotal varicocoele may have precipitated rupture of other portal-systemic bypasses. We consequently advocate cautious assessment of possible hydrocoele in patients with portal hypertension.

## Consent

Written informed consent was obtained from the patient for publication of this case report and any accompanying images. A copy of the written consent is available for review by the Editor-in-Chief of this journal.

## Competing interests

The authors declare that they have no competing interests.

## Authors' contributions

GY prepared the manuscript. AR and LB undertook the ultrasound examinations. NB instigated the report and critiqued the manuscript.
